# Systematic review and meta-analysis of the effectiveness of moxibustion therapy for primary dysmenorrhea

**DOI:** 10.3389/fmed.2025.1545146

**Published:** 2025-02-19

**Authors:** ShiWei Song, Hao Chen

**Affiliations:** ^1^Department of Traditional Chinese Medicine, Sichuan Taikang Hospital, Chengdu, China; ^2^Chengdu University of Traditional Chinese Medicine CN, Chengdu, China

**Keywords:** moxibustion, primary dysmenorrhea, systematic review, meta-analysis, randomized controlled trials

## Abstract

**Background:**

Primary dysmenorrhea is a common gynecological disease. Compared with traditional Chinese medicine treatment, moxibustion has advantages as a main treatment method. Therefore, we conducted a systematic review and meta-analysis to evaluate the efficacy of simple moxibustion therapy for primary dysmenorrhea.

**Methods:**

Randomized controlled trials were searched from PubMed, Web of Science, Embase, The Cochrane Library, China National Knowledge Infrastructure, Wan-fang database and VIP database. In the literature included in these databases, clinical reporters evaluated the efficacy of moxibustion as the treatment for primary dysmenorrhea. All included literature was assessed for risk bias by using Risk of Bias assessment tool 2.0, and meta-analysis was conducted using Rev. Man 5.4.

**Results:**

The findings demonstrated that the moxibustion group exhibited a statistically significant response in comparison to the control group. The improvement observed in the Cox Menstrual Symptom Scale and the visual analogue scale score between the two groups exhibited heterogeneity, with a statistically significant difference noted. In terms of Traditional Chinese Medicine symptom scores, the experimental group demonstrated superiority over the control group. Furthermore, the progesterone levels in the moxibustion treatment were found to be higher than in the control group, while the estrogen levels in the experimental group were lower than in the control group, with a statistically significant difference observed (*p* < 0.05). Conversely, the levels of *β*-EP and PGE2 in the observation group were higher than those in the control group.

**Conclusion:**

Moxibustion therapy shows significantly better efficacy in treating primary dysmenorrhea. However, a large sample, multi-center, high-quality RCT is still needed to evaluate its safety and efficacy.

**Systematic review registration:**

https://www.crd.york.ac.uk/PROSPERO/, Identifier CRD42024580466.

## 1 Introduction

Dysmenorrhea is a condition characterized by spasmodic pain in the lower abdomen during or around a woman’s period (1). This disease can be divided into primary dysmenorrhea and secondary dysmenorrhea. Primary dysmenorrhea, also known as functional dysmenorrhea, occurs in adolescent and reproductive age women, the global incidence of more than 50%, or even up to 90%, is one of the most common gynecological diseases (2, 3). The incidence of dysmenorrhea in China is more than 30%, and the proportion of primary dysmenorrhea is as high as 50% (4). Young women are the main population of primary dysmenorrhea, most patients with dysmenorrhea symptoms last for years or even several years, some can develop to middle age. Zheng’s research (5) shows that about 11 percent of female college students need to take long-term painkillers to control their dysmenorrhea symptoms, and most patients spend an average of 20 yuan a month due to dysmenorrhea. Research on dysmenorrhea in the United States shows that it costs women about $2 billion a year in absences from work and economic losses (6). In the development of primary dysmenorrhoea, oestrogen exacerbates the symptoms of dysmenorrhoea by modulating the expression of inflammatory mediators and promoting the synthesis of prostaglandins; whereas a decrease in the level of *β*-EP, an endogenous analgesic substance, is closely associated with the aggravation of dysmenorrhoea. PGE2 and PGF2α, as inflammatory mediators, are directly involved in the pathological process of dysmenorrhoea. Therefore, modulating the levels and roles of these molecules may be important in the treatment of primary dysmenorrhoea. This treatment of dysmenorrhea with western medicine has brought huge economic burden and public health pressure to the society and the country. At the same time, Western medicine has obvious side effects, so it is urgent to find a complementary and alternative therapy to treat dysmenorrhea. Compared with conventional treatment, moxibustion has become a better choice for most women to relieve dysmenorrhea. Through numerous clinical trials, researchers have found that moxibustion has significant advantages in the treatment of primary dysmenorrhea. Traditional Chinese medicine, including moxibustion, has been widely used in the clinical treatment of dysmenorrhea, and has shown clear curative effect in related studies. In recent years, moxibustion, as a green therapy, has been favored by female patients for its painless, effective and small side effects in the treatment of primary dysmenorrhea. However, moxibustion is considered to be a treatment used alone, and most previous systematic reviews have evaluated the efficacy of moxibustion in combination with other treatments. Therefore, we conducted this meta-analysis by sifting through the literature in recent years to evaluate the efficacy of moxibustion therapy alone in improving primary dysmenorrhea symptoms. At the same time, it also provides strong evidence support for the treatment of primary dysmenorrhea.

## 2 Materials and methods

### 2.1 Search strategy

A complete protocol for this study has been registered at PROSPERO (no. CRD42024580466). Chinese databases include China National Knowledge Infrastructure, VIP database and Wan-fang database. Foreign language databases include Web of Science, PubMed, Embase and the Cochrane Library. The retrieval time is from the establishment of the database to 16 August, 2024. In addition, grey literature was searched manually. The keywords in the search include “moxibustion,” “acupuncture,” “needle warming moxibustion,” “moxa,” “wormwood,” “laser moxibustion,” “primary dysmenorrhea,” “dysmenorrhea,” “menstrual pain” and “painful period.” The searching strategy of PubMed is taken as an example, which is shown in Table 1.

**Table 1:** PubMed: session results.

Number	Search details	Results
#1	“Moxibustion”[Mesh]	2,951
#2	“Moxibustion”[Title/Abstract] OR “Acupuncture”[Title/Abstract] OR “moxa”[Title/Abstract] OR “wormwood”[Title/Abstract] OR “laser moxibustion”[Title/Abstract] OR “needle warming moxibustion”[Title/Abstract]	31,255
#3	#1 OR #2	31,336
#4	“Dysmenorrhea”[Mesh]	4,830
#5	((((dysmenorrhea[Title/Abstract]) OR (primary dysmenorrhea[Title/Abstract])) OR (functional dysmenorrhea[Title/Abstract])) OR (menstrual pain[Title/Abstract])) OR (painful period[Title/Abstract])	7,291
#6	#4 OR #5	8,638
#7	#3 AND #6	281
#8	((“Moxibustion”[MeSH Terms] OR (“Moxibustion”[Title/Abstract] OR “Acupuncture”[Title/Abstract] OR “moxa”[Title/Abstract] OR “wormwood”[Title/Abstract] OR “laser moxibustion”[Title/Abstract] OR “needle warming moxibustion”[Title/Abstract])) AND (“Dysmenorrhea”[MeSH Terms] OR (“Dysmenorrhea”[Title/Abstract] OR “primary dysmenorrhea”[Title/Abstract] OR “functional dysmenorrhea”[Title/Abstract] OR “menstrual pain”[Title/Abstract] OR “painful period”[Title/Abstract]))) AND (randomizedcontrolledtrial[Filter])	73

### 2.2 Inclusion criteria

(1) Participants: diagnosed with primary dysmenorrhea according to the Primary Dysmenorrhea Consensus Guidelines (7), regardless of color of skin, gender, race, and source of the patients.
(2) Interventions: pure moxa stick treatment.
(3) Comparisons: Chinese patent medicine, western medicine, or acupuncture were included.
(4) Outcomes: the effective rate; the visual analogue scale score; The Cox Menstrual Symptom Scale; Traditional Chinese Medicine symptom scores, etc.
(5) Study Design: randomized controlled trials (RCT) of moxibustion in the treatment of primary dysmenorrhea.
(6) Language: English and Chinese.

### 2.3 Exclusion criteria

(1) The diagnostic criteria are unclear.
(2) Moxibustion combined with other treatment methods.
(3) Research on reviews, animal experiments, letters, comments, case reports.
(4) Secondary dysmenorrhea.
(5) The literature data is incomplete.
(6) Loss of outcome indicator.
(7) Full text not available.
(8) Nonclinical randomized controlled trials.
(9) Master’s dissertation or Doctoral thesis.

### 2.4 Literature inclusion and data extraction

After the literature search is completed, all records are imported into EndNote X9 for classified management. Two researchers independently select literature based on the inclusion and exclusion criteria and cross-check each other’s work. After an initial screening based on the title and abstract, the full text can be downloaded. After reading and analyzing, a second round of screening is conducted to exclude papers that clearly do not match the content of this study. If there are different opinions during the screening process, we need to discuss to reach a consensus. Excel was used to make a statistical analysis of the basic data of the study.

### 2.5 Quality assessment

The quality of literature was evaluated by low risk, some concerns or high risk. Two reviewers independently conducted the risk of bias assessment using the latest recommended tool from the Cochrane Handbook, Risk of Bias assessment tool 2.0 (ROB 2) (8). Evaluation indicators include: the randomization process, deviations from the intended interventions, missing outcome data, measurement of the outcome, selection of the reported result and other bias.

### 2.6 Statistical analysis

Dichotomous data is represented by the Odds Ratio (OR), and continuous data is represented by the Mean Difference (MD) for effect size. The combined effect is estimated using hypothesis testing and the 95% Confidence Interval (CI). Heterogeneity tests are measured by both Cochran’s Q-test and the I^2^. If there is no significant heterogeneity (*p* ≥ 0.1, I^2^ ≤ 50%), a fixed-effect model is used; on the contrary, if there is significant heterogeneity (*p* < 0.1, I^2^ > 50%), a random-effects model is employed for analysis, If necessary, a sensitivity analysis should be performed. The main statistical processes include heterogeneity testing, sensitivity analysis, meta-analysis, and funnel plot analysis.

## 3 Results

### 3.1 Literature search results and study characteristics

**Figure 1:**
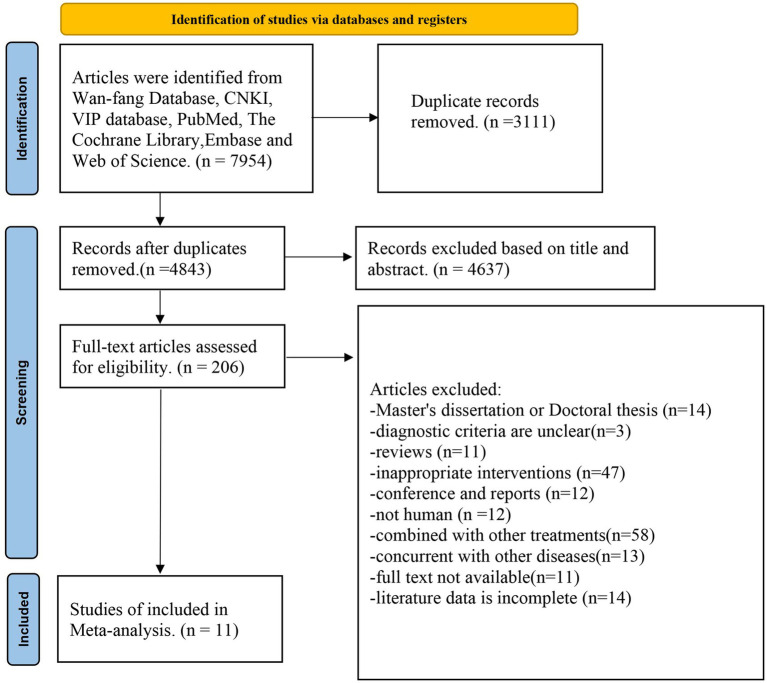
Flowchart of literature selection.

A total of 7,954 articles were searched and 3,111 duplicate references were excluded. By reading the abstract and title, 4,637 inappropriate articles were excluded, and 206 articles were further evaluated by reading them in full. A total of 195 articles were excluded, and it mainly includes the following contents: Master’s dissertation or Doctoral thesis (*n* = 14), diagnostic criteria are unclear (*n* = 3), reviews (*n* = 11); inappropriate interventions (*n* = 47), conference and reports (*n* = 12), not human (*n* = 12), combined with other treatments (*n* = 58), concurrent with other diseases (*n* = 13), full text not available (*n* = 11), literature data is incomplete (*n* = 14). Finally, a total of 11 studies were selected in the meta-analysis (Figure 1).

The basic features of the included literature were shown in Table 2 (9–19). A total of 11 articles were included in our study. The publication period of the articles is from 2010 to 2023. All studies included 918 patients, 459 patients in both the control group and the experimental group. Moxibustion therapy was used in 11 researches in the observation group, in the control group, 8 articles used drug therapy and 3 articles used acupuncture therapy. Basic information of the study included: the name of first author; study design; sample size; the number of cases; average age; duration of disorder; treatment period; adverse reaction; follow-up duration and outcome.

**Table 2:** Characteristics of 11 studies.

Study	Study design	Sample size (T:C)	Age (year), Mean ± SD	Disease duration (year), Mean ± SD	Intervention	Comparison	Treatment duration (days)	Follow-up (months)	Outcomes
Zhu et al. (19)	RCT	T:51 C:51	T:21.5士2.1 C:21.1士2.2	T:1.93士0.29 C:1.88士0.36	Moxibustion	Indometacin Enteric-coated Tablets, Oryzanol Tablets, Vitamin B1 tablets, Ibuprofen Sustained-release Capsules	3 menstrual cycles	NM	the effective rate, TCM
Huang et al. (17)	RCT	T:38 C:38	T:18.6士2.3 C:18.1士2.0	T:5.12士1.1 C:10.1士1.1	Moxibustion	Tong Jing Bao Ke Li	3 menstrual cycles	NM	the effective rate
Fang (10)	RCT	T:40 C:40	T:19.1士2.0 C:19.4士1.6	T:4.59士0.78 C:4.72士0.71	Moxibustion	Acupuncture	3 menstrual cycles	NM	the effective rate, COX
Wang and Chen (15)	RCT	T:60 C:60	T:22.47士2.92 C:22.07士2.31	T:6.67士3.95 C:6.41士2.55	Moxibustion	Ibuprofen Sustained-release Capsules	3 menstrual cycles	NM	the effective rate, VAS, COX
Bai et al. (9)	RCT	T:40 C:40	T:23.40士3.65 C:24.20士3.88	T:4.58士2.44 C:4.82士2.76	Moxibustion	Ibuprofen Sustained-release Capsules	3 menstrual cycles	NM	the effective rate, VAS, TCM
Jiang (12)	RCT	T:40 C:40	T:30.05士10.24 C:29.52士10.34	T:6.88士0.97 C:6.32士0.96	Moxibustion	Acupuncture	3 menstrual cycles	NM	the effective rate, progesterone, estrogen
Su and Zou (13)	RCT	T:30 C:30	T:32.12士1.15 C:32.16士1.28	T:2.54士1.07 C:3.05士1.38	Moxibustion	Acupuncture	3 menstrual cycles	NM	VAS, COX
Zeng (17)	RCT	T:40 C:40	T:19.94士3.20 C:20.02士3.23	T:2.47士0.92 C:2.40士0.78	Moxibustion	Tong Jing Bao Ke Li	3 menstrual cycles	NM	the effective rate, VAS, TCM
Zhao (18)	RCT	T:39 C:39	T:28.58士5.83 C:27.82士6.04	T:5.89士1.28 C:6.02士1.31	Moxibustion	Ibuprofen Sustained-release Capsules	3 menstrual cycles	NM	the effective rate, VAS, TCM
Wan et al. (14)	RCT	T:30 C:30	T:23士4.7 C:24士5.2	T:3.2士2.6 C:3.4士2.0	Moxibustion	Ibuprofen Sustained-release Capsules	3 menstrual cycles	NM	the effective rate, VAS, TCM
Wu and Xie (16)	RCT	T:51 C:51	T:25.61士2.70 C:25.83士2.99	T:4.41士0.85 C:4.23士0.79	Moxibustion	Ibuprofen Sustained-release Capsules	3 menstrual cycles	NM	the effective rate, VAS, COX, TCM, β-EP, PGE2, PGF2α

T, intervention group; C, comparison group; NM, not mention; RCT, randomized controlled trial; VAS, the visual analogue scale score; COX, The Cox Menstrual Symptom Scale; TCM, Traditional Chinese Medicine symptom scores.

10 studies evaluated the effective rate of the treatment, 7 articles analyzed the scores on the visual analogue scale score. Four RCTs measured outcomes using the Cox Menstrual Symptom Scale, while 6 articles focused on Traditional Chinese Medicine symptom scores. Additionally, one article reported on progesterone and estrogen levels, and another assessed a specific serum factor.

### 3.2 Inclusion of literature quality assessment

**Figure 2:**
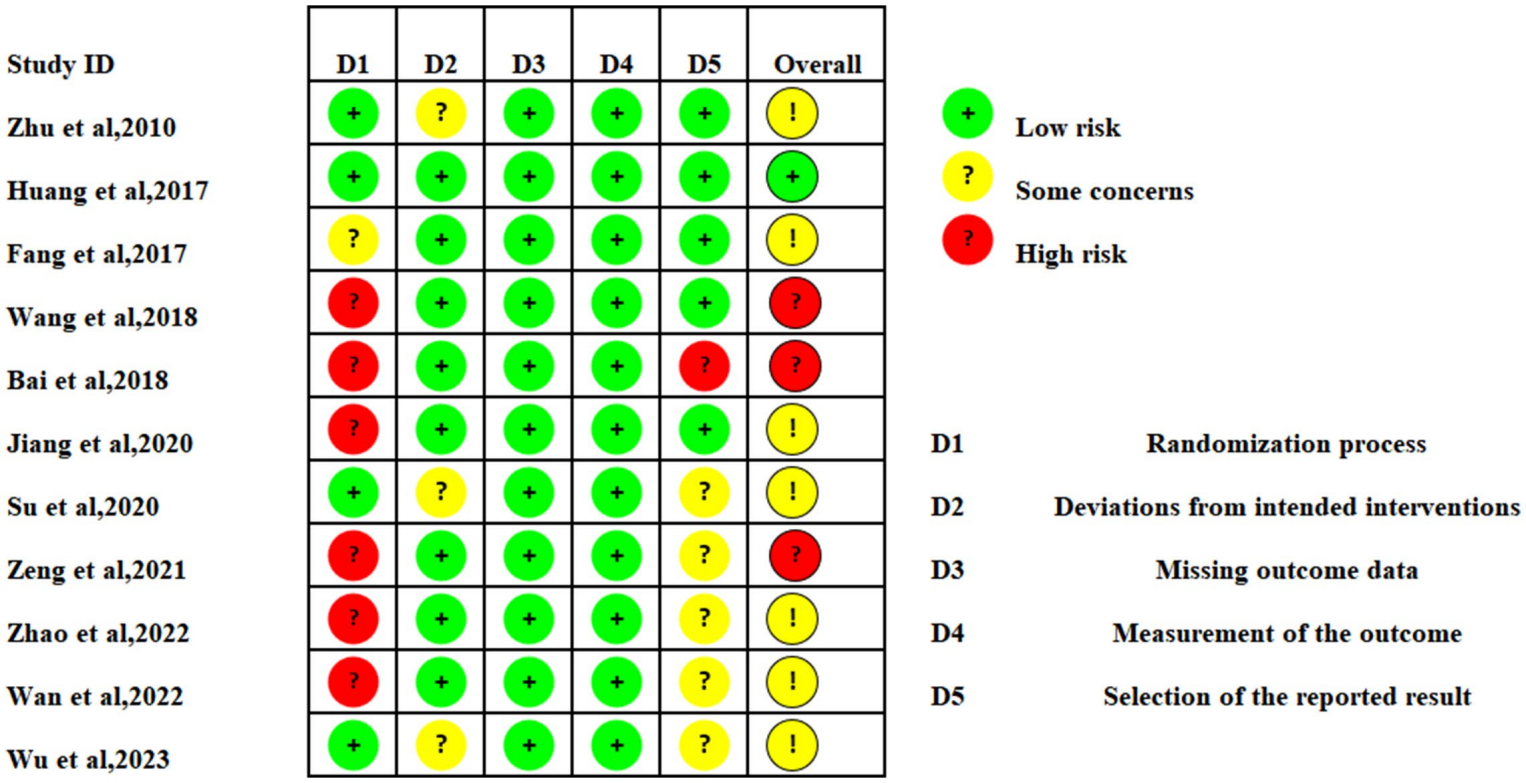
Assessment of risk of bias of 11 articles.

**Figure 3:**
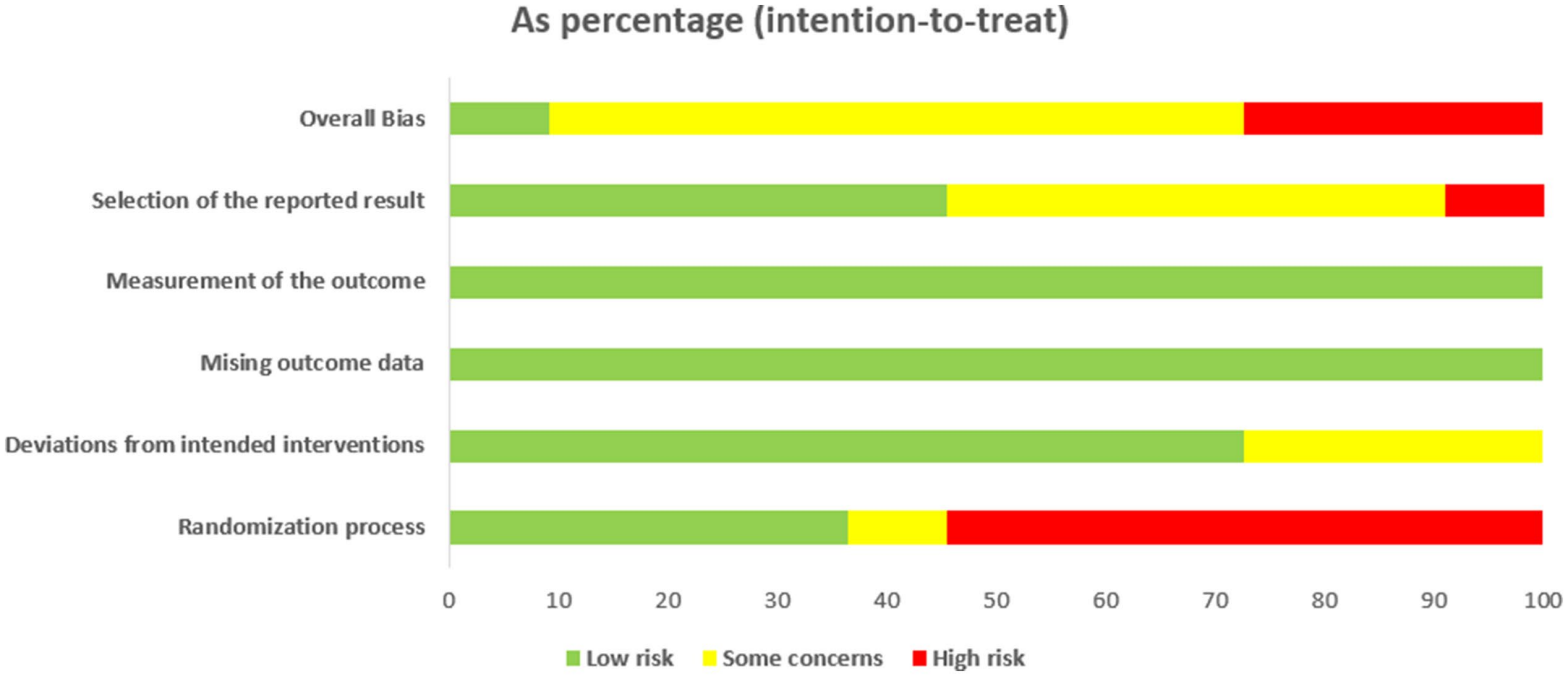
Assessment of risk of bias summary.

The overall quality of 11 literature studied was relatively low. 3 studies were at high risk of bias. 7 studies were considered to be at risk of “some concern,” and one study were at low risk. In the randomization process, 4 studies were at low risk, random number tables were commonly used. More than half the articles were considered “high risk,” because of the particularity of moxibustion, patients and researchers could not be blinded. For deviations from the intended interventions, 8 studies were at low risk, and others were considered to be at risk of “some concern.” All literature has complete outcome indicator data. In terms of outcome measurement, all studies are at low risk, and the measurement methods of the experimental group and the control group are comparable. A summary and graph are shown in Figures 2, 3.

### 3.3 The effective rate

**Figure 4:**
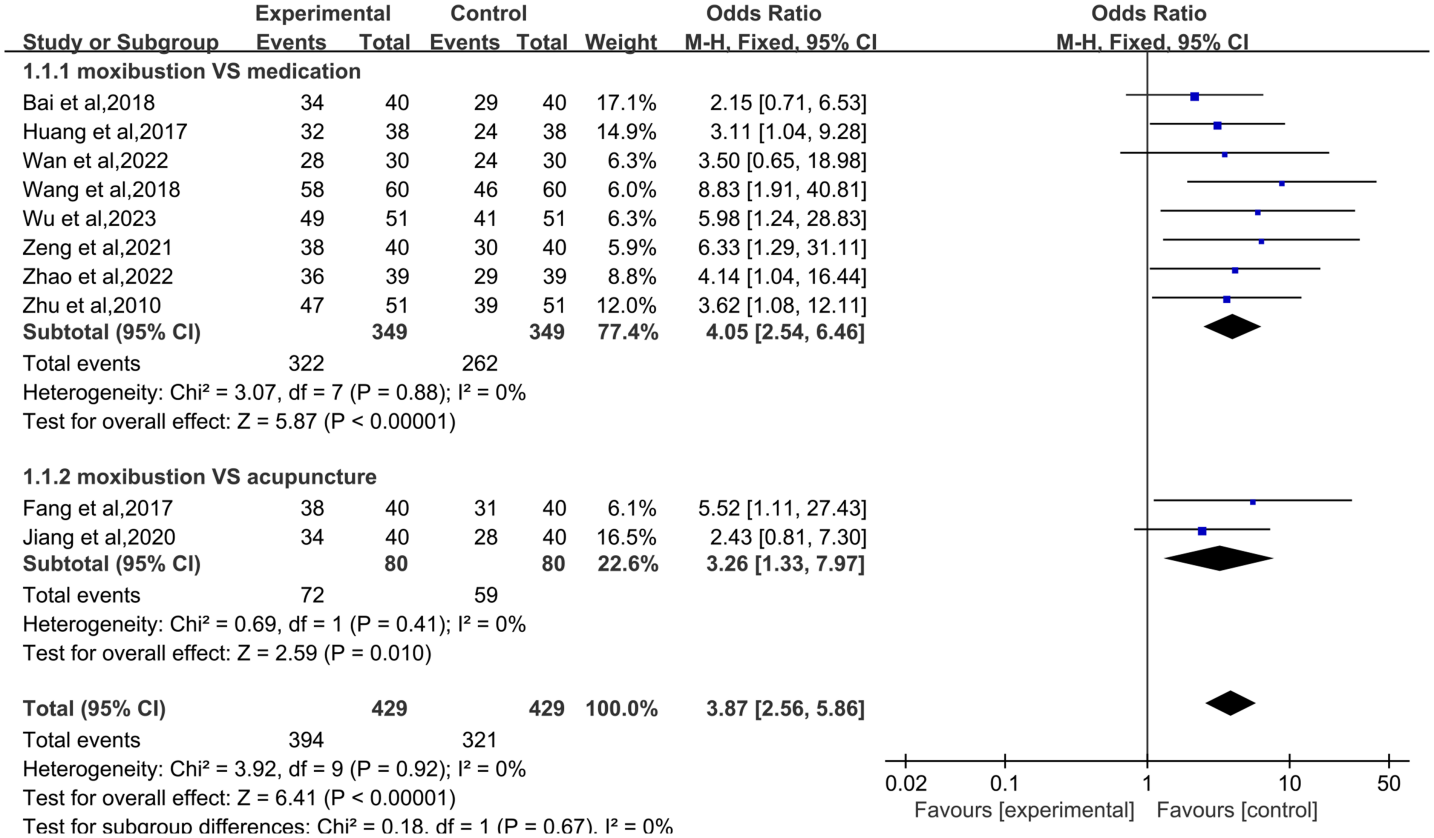
Forest plot of moxibustion efficiency for primary dysmenorrhea.

The effective rate was pooled for 10 studies (9–12, 14–19), a total of 858 patients were included, including 429 in both the experimental group and the control group. Heterogeneity analysis showed that there was obvious homogeneity among the studies, a fixed-effect model was employed (I^2^ = 0%; *p* = 0.92). The results showed that the total effective rate of moxibustion on primary dysmenorrhea was higher than that of control group (OR: 3.87; 95% CI: 2.56–5.86; *p* < 0.00001, Figure 4), the control group was divided into two subgroups according to different treatment plans. The results of subgroup analysis showed that the total effective rate of moxibustion in the treatment of primary dysmenorrhea was higher than that of medication alone (OR: 4.05; 95% CI: 2.54–6.46; *p* < 0.00001, Figure 4). The total effective rate of moxibustion is higher than that of acupuncture (OR: 3.26; 95% CI: 1.33–7.97; *p* = 0.010, Figure 4).

### 3.4 The visual analogue scale score

**Figure 5:**
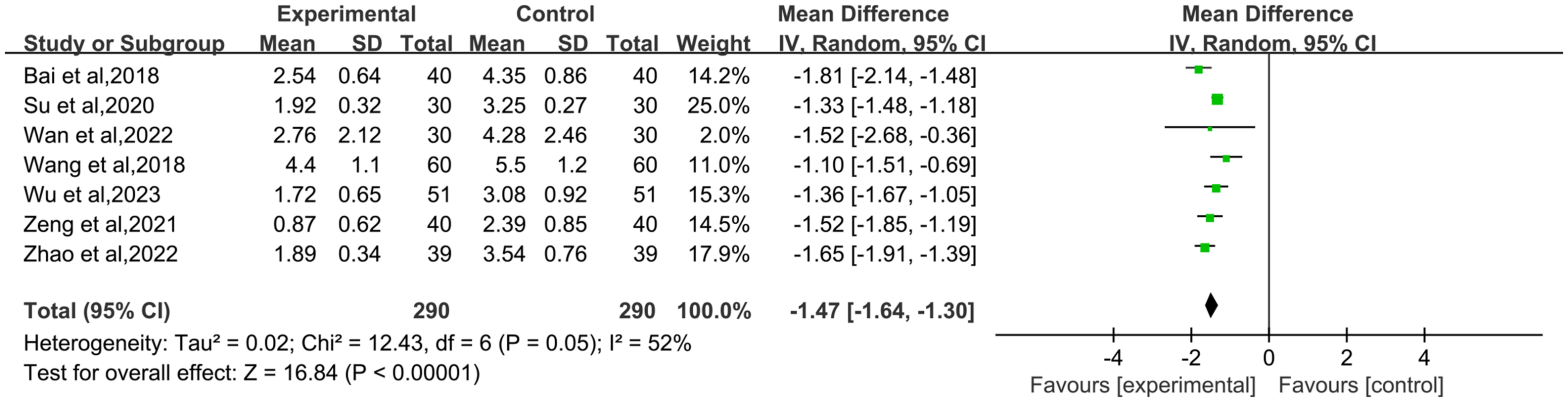
Forest plot of the visual analogue scale score for patient with primary dysmenorrhea.

7 studies (9, 13–18) used visual analogue scale score to assess treatment effects on pain. Heterogeneity among studies was medium strength, so random-effects model was selected (I^2^ = 52%; *p* = 0.05). In conclusion, the effect of moxibustion on reducing dysmenorrhea pain was better than that of control group (MD: −1.47; 95% CI: −1.64 – −1.30; *P* < 0.00001, Figure 5). In clinical practice, a reduction of 2 or more points in VAS score (e.g., from 6 to less than 4) is usually considered clinically significant pain relief. In our study, the mean change in VAS score was −1.47, which means that patients’ pain changed from “moderate pain” to “mild pain” or “no pain.” This means that patients’ pain changed from “moderate pain” to “mild pain” or “no pain.” Although this change is statistically significant, it may only be clinically relevant for some patients, especially those with high initial VAS scores.

### 3.5 The cox menstrual symptom scale

**Figure 6:**
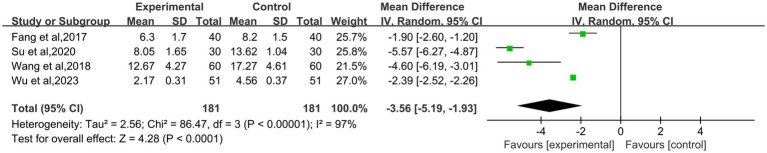
Forest plot of the cox menstrual symptom scale.

Four RCTs (10, 13, 15, 16) assessed the cox menstrual symptom scale. Heterogeneity analysis showed high heterogeneity among all studies (I^2^ = 97%; *P* < 0.00001). We analyzed the source of heterogeneity using the method of one by one elimination of references, and the result was robust. Meanwhile, our subgroup analysis based on the type of control intervention (drug versus acupuncture) revealed no significant change in heterogeneity, and we consider that the source of heterogeneity may be related to the acupuncture points and duration of moxibustion, as well as the patients’ subjective pain perception, so we chose the random effects model. The meta-analysis showed that moxibustion was more effective than the control group in decreasing the cox menstrual symptom scale (MD: −3.56; 95% CI: −5.19 – −1.93; *P*<0.0001, Figure 6).

### 3.6 Traditional Chinese medicine symptom scores

**Figure 7:**
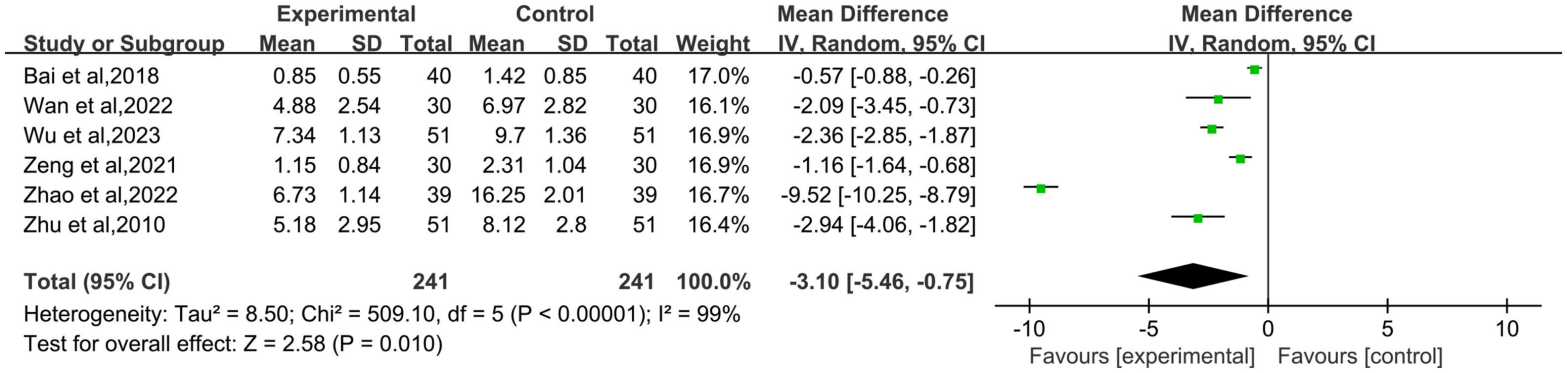
Forest plot of traditional Chinese medicine symptom scores in the treatment of primary dysmenorrhea.

The data about Traditional Chinese Medicine symptom scores in the treatment of primary dysmenorrhea were available in 6 studies (9, 14, 16–19). This combined statistics showed heterogeneity in Traditional Chinese Medicine symptom scores, so a random effects model was used (I^2^ = 99%; *P*<0.00001). The results showed that Traditional Chinese Medicine symptom scores of moxibustion group was lower than that of control group, and the difference was statistically significant (MD: −3.10; 95% CI: −5.46 – −0.75; *p* = 0.010, Figure 7).

### 3.7 Other evaluation indicators

1 article reported progesterone and estrogen (12), compared with the control group, the progesterone level in the moxibustion treatment was higher than the control group, while the estrogen level in the experimental group was lower than the control group. The difference was statistically significant (*p* < 0.05). Additionally, only 1 article measured the *β*-EP, PGE2, PGF2α (16). In the observation group, the levels of β-EP and PGE2 were higher than those in the control group, while the level of PGF2αwas lower than that in the control group (*p* < 0.05).

### 3.8 Safety assessment

In the 11 studies, only 3 articles (13, 16, 19) mentioned adverse reactions: Zhu et al. (19): control group: gastrointestinal reactions (15 cases), dizzy and headache (6 cases). Su and Zou (13) reported one case of burning sensation both in the treatment group and control group. Wu and Xie (16) showed that the in the western medicine group, two patient experienced gastrointestinal reaction, in the moxibustion group, there was one case each of blisters, red swelling, and itching on the skin, all of which did not affect the progress of subsequent research.

### 3.9 Publication bias

**Figure 8:**
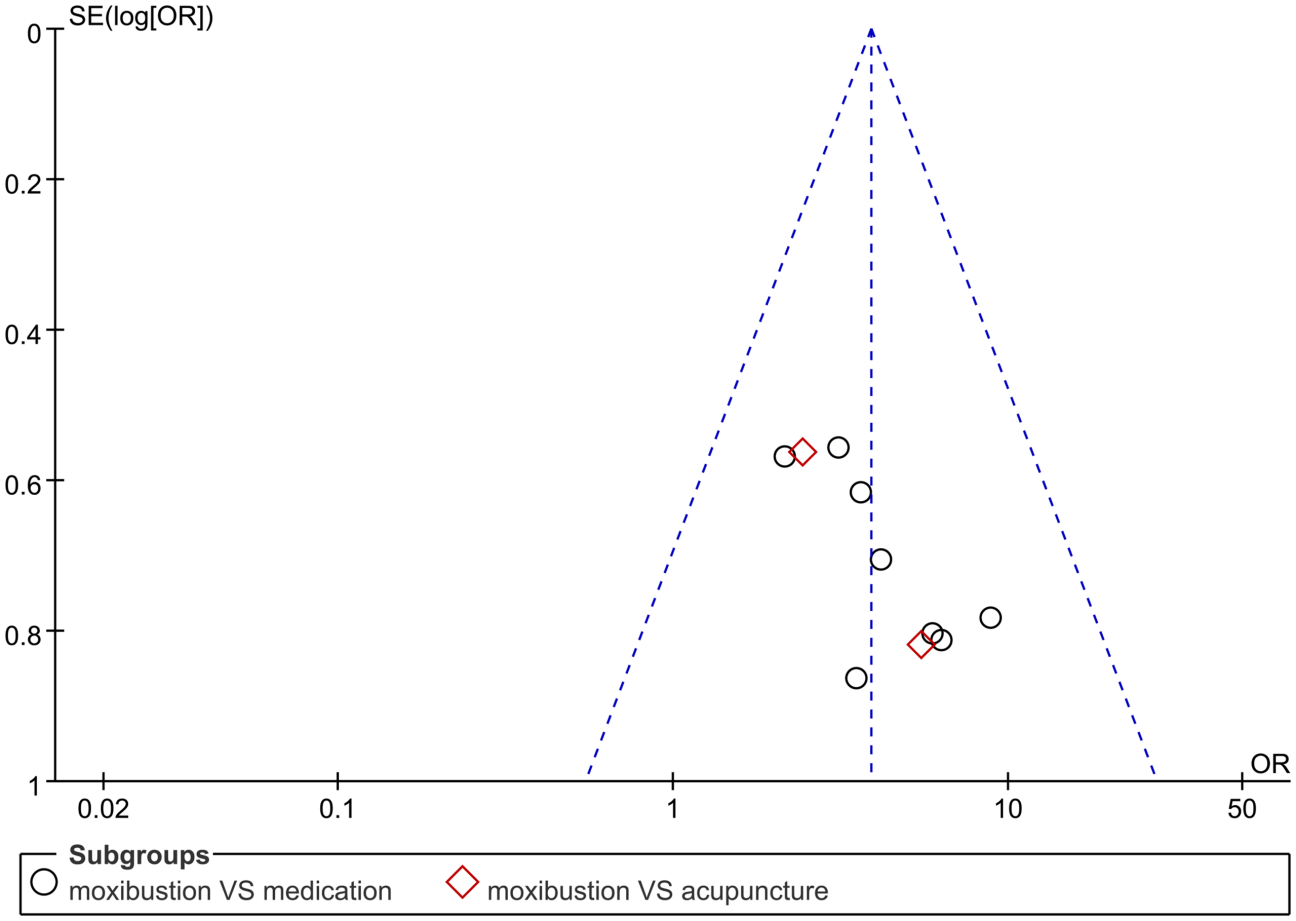
Funnel plot analysis of the effective rate.

This study used the effective rate as the outcome indicator and conducted an analysis of publication bias. The results of the publication bias analysis of the 10 included studies showed that most of the scatter points were located in the middle and lower part of the inverted triangle, and the distribution was relatively symmetrical on both sides, suggesting a small publication bias (Figure 8).

## 4 Discussion

### 4.1 Summary of main results

The study systematically assessed the outcome indicators of alleviating primary dysmenorrhea with single moxibustion therapy, including the effective rate, the cox menstrual symptom scale, Traditional Chinese Medicine symptom scores, the visual analogue scale score, progesterone estrogen, β-EP, PGE2 and PGF2. Eleven studies, encompassing a total of 918 patients with primary dysmenorrhea, were incorporated into this comprehensive analysis. The results underscore the significant enhancement of clinical therapeutic outcomes attributable to moxibustion. Concurrently, moxibustion has been shown to markedly alleviate the symptoms of dysmenorrhea.

In today’s society, with the increase in economic and life and work burdens, women are under tremendous physiological and psychological pressure. Their enthusiasm in all aspects is suppressed, and the fast-paced life, lack of physical exercise, changes in dietary habits, and staying up late and other unhealthy lifestyles have led to an increasing incidence of primary dysmenorrhea (20). Woman is more sensitive and have a strong tolerance for mild pain, but they often lack health awareness. Over time, as the pain intensifies, the difficulty of treating primary dysmenorrhea increases, bringing a series of public health burdens (21). At present, it is openly acknowledged worldwide that the pathogenesis of primary dysmenorrhea is characterized by abnormally high levels of prostaglandins and interleukins (22). Prostaglandins cause a reduction in the relaxation of uterine smooth muscle, leading to spasms, which in turn result in a decrease in uterine blood flow. This also increases the sensitivity of the patient’s peripheral nerves to pain, ultimately causing dysmenorrhea (23). Further research has shown that primary dysmenorrhea is also related to factors such as endocrine disorders, abnormal function of vascular endothelial cells, neurotransmitters, and psychological and emotional factors (24). Sun’s research (25) found that factors such as oxytocin, vasopressin, estradiol, endothelin, and beta-endorphin also have an important association with the occurrence of primary dysmenorrhea.

In the present social era, modern medicine does not have a clear treatment method for primary dysmenorrhea and the main measure is painkillers (7). In clinical guidelines for dysmenorrhea, nonsteroidal anti-inflammatory drugs (NSAIDs) and hormonal contraceptives are recommended as first-line medications for treating dysmenorrhea, with other drugs including calcium channel blockers (26). However, Western medicine treatment has a high recurrence rate and obvious adverse reactions in the gastrointestinal and nervous systems, and there is a certain failure rate (27). For stubborn and refractory primary dysmenorrhea, surgical therapy may be used when necessary, with common surgical methods including nerve resection and cervical dilation (28, 29). Both Western medicine treatment and surgical treatment have side effects, and inevitably cause some damage to physical and mental health.

Therefore, it is necessary to explore the necessity of alternative or supplementary comprehensive treatments for primary dysmenorrhea (30). Based on this, moxibustion has a broader development space and prospects. Moxibustion, as a traditional and ancient Chinese medical external treatment method, has many advantages, such as being affordable, painless, free of side effects, and having a definite therapeutic effect, as well as playing a role in health preservation and health care (31). In recent years, research has shown that moxibustion can promote changes in hemorheology, alleviate dysmenorrhea by increasing uterine blood flow, reducing blood viscosity, inhibiting uterine smooth muscle contractions, and improving uterine microcirculation, thereby exerting an analgesic effect (32, 33).

### 4.2 Analysis of strengths and limitations

This study analyzes clinical studies on the treatment of primary dysmenorrhea with moxibustion. Among the 11 included literatures, 10 are clinical observation documents from the past 7 years. The treatment group uses an independent moxibustion therapy, while the control group chooses either solo drug treatment or acupuncture therapy, which is rare in previous studies. This study demonstrates that moxibustion, as a primary traditional Chinese external treatment method, has a significant effect on the treatment and health care of women’s dysmenorrhea. The conclusions drawn have certain reference value.

Furthermore, our study also has some limitations. Firstly, the literature included in this meta-analysis is relatively homogenous in language, limited to Chinese, and the quality of Chinese randomized controlled trials varies, which introduces language bias and is not conducive to the generalization of the research. Secondly, the publication quality of this study is average, with all literature not emphasizing the use of the double-blind method. Thirdly, this study included 11 articles, with the largest sample size being only 120 cases, lacking large-sample clinical data. There are a limited number of studies on the effects of moxibustion treatments on progesterone and estrogen levels. Only one report mentions that moxibustion may have a positive effect on these hormone levels. Therefore, we emphasise that this conclusion is based on a limited number of reported cases and that more studies are needed for further validation. Fourthly, the selection of acupoints, operation techniques, duration, and stimulus amount in various moxibustion methods are not the same, and there is individual variability in treatment, leading to subjectivity in pain symptom scoring. These factors have to some extent affected the accuracy of the conclusions of this study. The source of heterogeneity may be due to differences in patients’ subjective pain sensations, as well as in moxibustion point selection, technique, and duration of moxibustion. Continuous optimisation of the study protocol is needed in future studies.

## 5 Conclusion

To sum up, this study proved that moxibustion alone has obvious efficacy in relieving primary dysmenorrhea by analyzing the effectiveness and safety of moxibustion in treating primary dysmenorrhea. It is hoped that in future studies, reasonable literature screening criteria and literature search strategies can be formulated, meta methods can be scientifically and accurately applied, and more high-quality randomized controlled trials with multi-center, large samples can be conducted to provide a more objective evaluation of moxibustion in the treatment of primary dysmenorrhea. In the meantime, we suggest that future studies could further explore the dose-effect relationship of different strengths of moxibustion, as well as focus on the selection of empirical acupoints for the treatment of primary dysmenorrhoea, in order to develop a more precise and standardized protocol.

There have been studies on spatial transcription technology. High-plex protein and whole transcriptome co-mapping at cellular resolution with spatial CITE-seq, Spatial dynamics of mammalian brain development and neuroinflammation by multimodal tri-omics mapping, Spatially Resolved *in vivo* CRISPR Screen Sequencing via Perturb-DBiT (34). These techniques not only reveal the spatial distribution of cell types and gene expression, but also provide insight into the role of gene regulatory networks and gene function in the tissue microenvironment. It may provide a more precise basis for future studies of moxibustion in the treatment of primary dysmenorrhoea.

## Data Availability

The original contributions presented in the study are included in the article/supplementary material, further inquiries can be directed to the corresponding author.
